# Differential Immune Priming Effects of Banana Extracellular Self-DNA Derived from Bananas with Varying Disease Severities Against *Fusarium oxysporum* f. sp. *cubense* Tropical Race 4

**DOI:** 10.3390/jof12060438

**Published:** 2026-06-15

**Authors:** Yuxuan Hu, Dandan Wei, Junyou Wang, Jinku Li, Pingshan Fan, Yunze Ruan

**Affiliations:** 1 College of Tropical Agriculture and Forestry, Hainan University, Danzhou 571737, China; hyxhainanu@126.com (Y.H.); lljinku@126.com (J.L.); 2 School of Breeding and Multiplication (Sanya Institute of Breeding and Multiplication), Hainan University, Sanya 572025, China; hnuwdd@126.com (D.W.); huwangjunyou@163.com (J.W.)

**Keywords:** extracellular self-DNA, banana *Fusarium* wilt, disease-stage-dependent immunity, transcriptome, plant–pathogen interaction

## Abstract

Banana *Fusarium* wilt, caused by *Fusarium oxysporum* f. sp. *cubense* tropical race 4 (Foc TR4), poses a significant threat to global banana production; however, effective and sustainable control strategies remain limited. Extracellular self-DNA (esDNA), which functions as a damage-associated molecular pattern (DAMP), has recently been identified as a crucial regulator of plant innate immunity. Nonetheless, it is unclear whether the immune regulatory function of esDNA varies with disease progression. In this study, we examined the effects of esDNA derived from banana leaves exhibiting different disease severities on plant resistance to *Fusarium* wilt. Hydroponic experiments revealed that esDNA displayed a distinct disease-stage-dependent regulatory pattern. EsDNA from mildly diseased tissues significantly suppressed Foc TR4 colonization, supported plant growth, and mitigated oxidative damage, whereas esDNA from severely dise ased tissues lost protective effects and even intensified cellular stress. Physiological analyses indicated that beneficial esDNA effectively reduced H_2_O_2_ and malondialdehyde accumulation while enhancing antioxidant enzyme activities and phenylpropanoid metabolism. Transcriptome profiling further demonstrated that esDNA extensively altered pathogen-induced gene expression, with enrichment of pathways involved in metabolic and redox homeostasis. These transcriptional changes correlate with the observed reduction in oxidative damage and improved plant growth, suggesting that restoration of homeostasis may contribute to esDNA-mediated resistance. Our findings collectively demonstrate that esDNA serves as a dynamic DAMP signal, exhibiting effects that depend on the disease stage. This study offers new insights into the role of plant self-DNA in mediating immunity and presents a promising strategy for developing environmentally sustainable control measures against banana *Fusarium* wilt.

## 1. Introduction

Banana (*Musa* spp.) is one of the most economically important tropical cash crops worldwide, playing an irreplaceable role in food security and economic stability across tropical and subtropical regions. However, *Fusarium* wilt caused by *Fusarium oxysporum* f. sp. *cubense* tropical race 4 (Foc TR4) has emerged as a severe threat to the global banana industry [1,2]. As a soil-borne vascular disease, Foc TR4 survives in soil for long periods as dormant chlamydospores. After root infection, the pathogen quickly invades and moves upward through vascular tissues, where it secretes cell wall-degrading enzymes and mycotoxins that cause vascular blockage and systemic dysfunction, ultimately producing leaf yellowing, pseudostem longitudinal cracking, and plant death [3,4,5]. Current management of Foc TR4 faces major challenges. Chemical fungicides poorly penetrate vascular tissues and can select for resistant strains. Conventional breeding for resistance is constrained by long generation times and limited genetic diversity. Biological control agents often display poor colonization and inconsistent efficacy under complex field conditions [2,6]. Thus, a detailed understanding of host immune responses and the development of novel, environmentally friendly control strategies that harness plant innate immunity are urgent priorities.

Recent work on plant innate immunity has highlighted the central role of damage-associated molecular patterns (DAMPs) in pattern-triggered immunity (PTI) [7,8,9], and, in particular, extracellular self-DNA (esDNA) released from host cells after stress or damage has been identified as a key DAMP signal [10,11]. Studies in model species—*Arabidopsis thaliana* [12], common bean [13,14,15]—show that esDNA rapidly triggers intracellular Ca^2+^ influx, a burst of reactive oxygen species (ROS), activation of MAPK cascades, and systemic induction of defense-related genes, thereby priming immunity or directly conferring resistance [16,17,18,19]. Most studies of esDNA-induced immunity employ purified DNA from healthy tissues or genomic DNA from a single pathogen as the stimulant, which oversimplifies how esDNA is generated and altered during natural infections. In soil-borne diseases, the extracellular DNA released during plant–pathogen interactions is likely a dynamic mixture that contains both “self” signals from damaged host cells and “non-self” signals released during pathogen proliferation, lysis, or death [16]. As disease progresses, this mixed eDNA can change markedly in fragment length distribution, cytosine methylation, and oxidative modifications [12,14,20,21]. How these chemical modifications and the increasing signal complexity of esDNA across pathological stages influence plant DAMP recognition, signal transduction, and ultimately resistance phenotypes—particularly in the highly specific banana–Foc TR4 pathosystem—remains largely unexplored and lacks systematic study [15,19].

To address these gaps, this study systematically examined how esDNA from banana tissues with varying disease severities differentially regulates banana resistance to *Fusarium* wilt. We addressed three key questions. First, does banana esDNA produce stable immune-inducing or resistance-priming effects under Foc TR4 stress? Second, do these effects vary according to the disease severity of the esDNA source? Third, if such variation exists, what are the underlying physiological and molecular mechanisms? Guided by disease dynamics and DAMP theory, we hypothesized that as disease severity increases, esDNA released from infected leaves undergoes progressive fragmentation and chemical modification, altering its recognition and signaling in recipient plants. Specifically, esDNA from healthy or mildly affected plants is predicted to act as an immune alert, modestly enhancing host defenses without imposing excessive stress; conversely, esDNA from severely diseased plants may lose protective potency because of extensive degradation or the presence of deleterious signals and might even exacerbate oxidative damage or provoke maladaptive depletion of immune–metabolic resources.

To test this hypothesis, we performed a controlled hydroponic experiment. Total DNA was extracted from banana leaves with different disease severities, sonicated to fragment the DNA and simulate the esDNA mixture released from damaged tissues [22], and then applied exogenously to recipient banana seedlings after concentration normalization. The study combined morphological phenotyping, physiological and biochemical assays, and molecular detection, and it used transcriptome sequencing (RNA-seq) to resolve global transcriptional responses induced by esDNA from different sources and to identify key pathways and hub genes. Theoretically, this work broadens the scope of DAMP signaling in soil-borne vascular disease systems. Practically, it provides a molecular foundation and strategic guidance for developing precise, green control strategies against banana *Fusarium* wilt based on self-DNA-induced immune priming [8,23,24].

## 2. Materials and Methods

### 2.1. Plant and Pathogen Materials

Banana plantlets used in this study were tissue-cultured ‘Brazil’ banana seedlings purchased from the Tissue Culture Center of the Haikou Experimental Station, Chinese Academy of Tropical Agricultural Sciences (Haikou, China). The pathogen Foc TR4 was isolated from rhizosphere soil of diseased banana plants in our laboratory. After identification by morphological characteristics and ITS rRNA sequencing, the strain was stored in a glycerol tube at −80 °C for later use.

### 2.2. Extraction and Processing of esDNA

Banana leaves with different *Fusarium* wilt severities (disease indices: 30%, 61%, 81%, 87%) were collected, snap-frozen in liquid nitrogen, and ground into a fine powder. Total DNA was extracted using a high-efficiency plant genomic DNA extraction kit (Tiangen, Beijing, China, DP304), and concentration was determined using a NanoDrop 2000 spectrophotometer (Thermo Scientific, Waltham, MA, USA). According to the method of [22], DNA was fragmented using 0.5 mm glass beads and an ultrasonic processor, and fragment size was verified by 1% agarose gel electrophoresis.

### 2.3. Preparation and Inoculation of Foc TR4 Spore Suspension

Foc TR4 mycelia activated on PDA plates for 3 days were inoculated into PDB liquid medium and incubated at 28 °C with shaking at 150 rpm for 48 h. After incubation, the mycelial precipitate was collected by centrifugation at 8000 r/min at 4 °C for 10 min and washed twice with sterile water [25,26,27]. After filtering out mycelial clumps through four layers of sterile gauze, the spore concentration was adjusted to 1.28 × 10^8^ cfu/mL using a hemocytometer and stored at 4 °C. We used the root-pricking inoculation method: a 0.5 mm sterile needle was used to obliquely puncture the banana root surface at a depth of 1 cm and intervals of 2 cm, with five wounds per plant. Then, 16 mL of Foc TR4 spore suspension (1 × 10^7^ cfu/mL) was applied to induce disease development [28,29].

### 2.4. Hydroponic Experimental Design

Healthy tissue-cultured seedlings with uniform growth and no pests or diseases were selected for the experiment. Before transplanting, roots were rinsed three times with sterile water to completely remove the medium. Seedlings were fixed with sponges at the base and placed in customized hydroponic boxes supplied with 1/10-strength Hoagland’s nutrient solution, which was renewed every 7 days. After pre-culture for 45 days, disease symptoms were observed [30]. Six treatments were applied 14 days after transplanting: CK: 16 mL sterile water (no pathogen, no esDNA); Foc: 16 mL Foc TR4 spore suspension; T1–T4: 5 μL esDNA solution (25 ng/μL) from corresponding disease levels, applied 5 days after Foc inoculation. Each treatment included four biological replicates. At 30 days after treatment, plant height and pseudostem diameter were measured. Shoots and roots were carefully separated, rinsed 2–3 times with deionized water, and fresh weights of different tissues were recorded. Part of the samples were stored at −20 °C, and the other part was fixed at 105 °C for 30 min and then dried to constant weight at 75 °C for dry weight measurement.

### 2.5. Root Morphology Analysis

Intact roots stored at 4 °C were placed in a transparent root tray, and lateral roots were gently spread using tweezers. Root images were scanned using an Epson Perfection V800 scanner (Epson, Los Alamitos, CA, USA), and total root length, mean root diameter, root surface area, and root volume were analyzed using the WinRHIZO system (Regent Instruments Canada Inc., Quebec City, QC, Canada) [31,32]. Three biological replicates were analyzed, and each root system was scanned once.

### 2.6. Assays of Resistance-Related Enzyme Activities and Proline Content

All enzyme activities and physiological indexes were determined using commercial kits (mlbio, Shanghai Enzyme-linked Biotechnology Co., Ltd., Shanghai, China) and measured on a SuPerMax 3100 microplate reader (Shanghai Flash Spectrum Biotechnology Co., Ltd., Shanghai, China). H_2_O_2_ content was determined by the titanium yellow colorimetric method; CAT activity by the ultraviolet absorption method at 240 nm; POD activity by the guaiacol oxidation method at 470 nm; SOD activity by the NBT photoreduction method at 560 nm; PAL activity by the L-phenylalanine deamination method at 290 nm; and proline (Pro) content by the acid ninhydrin method at 520 nm. For each treatment, three biological replicates were used. Each biological replicate was measured in three technical replicates; the mean value of the three technical replicates was used for statistical analysis.

### 2.7. Quantification of Foc TR4 Abundance

Root and corm samples (0.1 g) were collected 30 days after treatment, immediately frozen in liquid nitrogen, and finely ground. Total genomic DNA was extracted using a plant genomic DNA extraction kit (Tiangen, DP305), according to the manufacturer’s instructions. The concentration and purity of the extracted DNA were determined using a NanoDrop 2000 spectrophotometer (Thermo Fisher Scientific, Waltham, MA, USA). The abundance of Foc TR4 was quantified in triplicate by real-time quantitative PCR (qPCR) using a qTOWER3 Real-Time PCR System (Analytik Jena AG, Jena, Germany). The pathogen-specific primer pair used was FOF1 (5-ACATACCACTTGTTGCCTC-3′) and FOR1 (5-CGCCAATCAATTTGAGGAACG-3′) [33]. Each qPCR reaction (10 μL) contained 5 μL of 2× SYBR Premix Ex Taq (TaKaRa, Beijing, China), 0.5 μL each of forward and reverse primers (10 μmol L^−1^), 3 μL of template DNA (5–20 ng μL^−1^), and 1 μL of sterile deionized water. The thermal cycling conditions consisted of an initial denaturation at 95 °C for 5 min, followed by 40 cycles of denaturation at 95 °C for 30 s, annealing at 60 °C for 30 s, and extension at 72 °C for 30 s. A standard curve was generated using a 10-fold serial dilution series (10^1^–10^6^ copies) of a recombinant plasmid (FOC4-383; Wcgene Biotechnology, Shanghai, China) containing the internal transcribed spacer (ITS) region of Foc. The total plasmid length was 3093 bp, with an ITS target fragment of approximately 250 bp. Sterile deionized water was used as the no-template negative control. The amplification efficiency was 99.2%, with a correlation coefficient (R^2^) of 0.996. For each treatment, three biological replicates (individual plants) were analyzed, and each biological replicate included three technical replicates. The mean Ct value of the technical replicates was used to calculate the ITS gene copy number based on the standard curve.

### 2.8. Transcriptome Sequencing and Analysis

Leaf samples for transcriptome analysis were collected from the hydroponic experiment described in Section 2.4. Three treatments were included: CK (sterile water control), Foc (Foc TR4 inoculation alone), and Foc+esDNA (30%) (Foc TR4 inoculation combined with esDNA extracted from banana leaves with a disease index of 30%). esDNA was applied 5 days post Foc TR4 inoculation, with each plant receiving 5 μL of esDNA solution at 25 ng/μL.

Leaf samples were collected 30 days after treatment. Three independent biological replicates were collected per treatment, with each replicate consisting of pooled leaf tissue from three individual plants. Samples were immediately frozen in liquid nitrogen and stored at −80 °C.

Total RNA extraction, quality control, library construction, and sequencing were performed by Novogene Co., Ltd. (Beijing, China) using the Illumina NovaSeq 6000 platform [34]. High-quality clean reads were mapped to the banana reference genome (downloaded from NCBI) using Hisat2 (v2.0.5). For transcriptome analysis, three biological replicates were collected per treatment. Each biological replicate consisted of pooled leaf tissue from three individual plants to minimize biological variation. RNA extraction and sequencing were performed on each biological replicate separately; no technical replicates were included due to the high reproducibility of the sequencing platform.

### 2.9. Statistical Analysis

Statistical analysis was performed using SPSS 26.0 software. One-way analysis of variance was conducted to compare means among multiple groups. When ANOVA indicated significant differences, multiple comparisons were carried out using Tukey’s honestly significant difference test or Duncan’s multiple range test at a significance level of α = 0.05. Results are presented as means ± standard deviation or standard error, as indicated. For correlation analyses, Pearson or Spearman correlation coefficients were calculated, with *p* < 0.05 considered statistically significant.

For transcriptome data, differentially expressed genes (DEGs) were identified using the DESeq2 package in R (Version 1.0.13) with thresholds of adjusted *p* ≤ 0.01 and |log_2_FC| ≥ 1 [35]. Gene Ontology enrichment analysis was performed using the clusterProfiler package in R. The hypergeometric test was applied to identify significantly enriched GO terms among the DEGs. A threshold of adjusted *p*-value < 0.05, calculated using the Benjamini–Hochberg method, was considered statistically significant. The GO annotation reference database for Musa acuminata was downloaded from NCBI. KEGG pathway enrichment analysis was similarly performed using clusterProfiler against the Musa acuminata KEGG reference background, with the same statistical threshold. Principal component analysis was used to visualize sample expression profiles [36,37], and heatmaps were generated using the pheatmap package (version 4.6.0) to reveal expression patterns and regulatory mechanisms.

## 3. Results

### 3.1. Effects of esDNA on Banana Growth and Root Morphology

**Figure 1:**
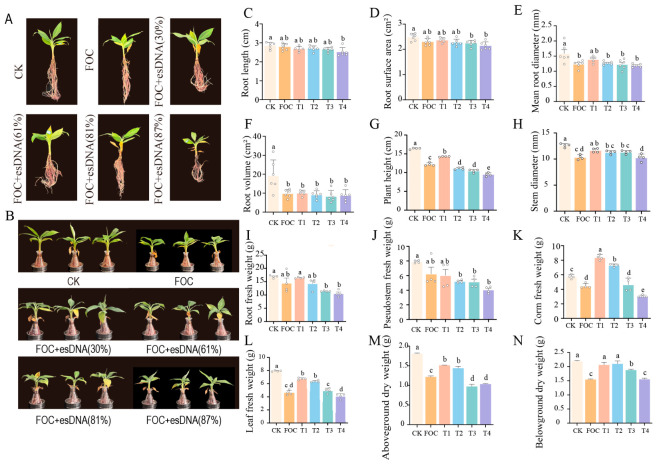
Phenotypic and biomass responses of banana plantlets to Foc TR4 infection following exogenous extracellular self-DNA (esDNA) application. (**A**,**B**) Representative phenotypes of whole plants and root systems under the non-inoculated control (CK), Foc TR4 inoculation alone (Foc), and Foc TR4 inoculation combined with esDNA extracted from leaves with disease indices of 30%, 61%, 81%, and 87% (T1: FOC+esDNA (30%), T2: FOC+esDNA (61%), T3: FOC+esDNA (81%), T4: FOC+esDNA (87%)). (**C**–**F**) Quantification of root architectural traits: root length (**C**), root surface area (**D**), mean root diameter (**E**), and root volume (**F**). (**G**,**H**) Agronomic traits of the aboveground part: plant height (**G**) and pseudostem diameter (**H**). (**I**–**L**) Tissue-specific fresh biomass: leaf fresh weight (**I**), pseudostem fresh weight (**J**), root fresh weight (**K**), and corm fresh weight (**L**). (**M**,**N**) Dry biomass partitioning: aboveground dry weight (**M**) and belowground dry weight (**N**). Values are means ± SD or SE. Different lowercase letters indicate significant differences among treatments (one-way ANOVA followed by Duncan’s multiple range test, *p* < 0.05).

Both exogenous esDNA application and pathogen inoculation significantly inhibited banana root growth. Total root length, surface area, mean diameter, and total volume differed significantly among treatments (*p* < 0.05). The control (CK) exhibited the highest values for all root indices, while esDNA from severely diseased tissues (87%) produced the strongest inhibitory effect (Figure 1A,C–F). Overall, compared with CK, all esDNA treatments and Foc inoculation restricted root elongation, reduced surface area, decreased diameter, and lowered total volume. Aboveground growth traits showed tissue-specific responses to esDNA that contrasted with the general root inhibition. Pseudostem diameter and fresh weights of leaves and roots remained highest in CK. However, esDNA from mildly and moderately diseased leaves (30% and 61%) conferred relative advantages in plant height and corm fresh weight, which were significantly greater than those observed with Foc inoculation and with esDNA from severely diseased tissues (Figure 1B,G–N).

### 3.2. Inhibitory Effects of esDNA from Different Sources on Foc Proliferation in Banana Corm and Root

**Figure 2:**
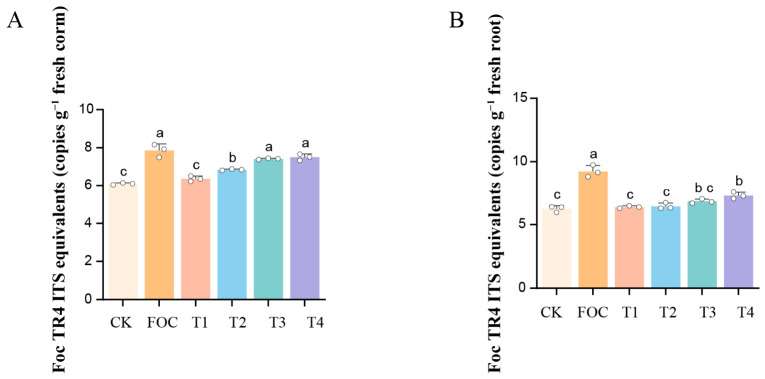
Effects of exogenous extracellular self-DNA (esDNA) derived from banana leaves with varying disease severities on *Fusarium oxysporum* f. sp. *cubense* tropical race 4 (Foc TR4) abundance in banana corm and root tissues. (**A**,**B**) Quantitative analysis of relative Foc TR4 abundance expressed as ITS copy number equivalents per gram of fresh tissue in corm (**A**) and root (**B**) determined by qPCR. Values represent ITS-amplicon equivalent copies calculated from a FOC4-383 plasmid-derived standard curve; these equivalents are used only for relative comparison across treatments rather than absolute genomic quantification of Foc TR4. Treatments: non-inoculated mock control (CK), Foc TR4 inoculation alone (Foc), and Foc TR4 inoculation supplemented with esDNA from leaves with disease indices of 30%, 61%, 81%, and 87% (T1: FOC+esDNA (30%), T2: FOC+esDNA (61%), T3: FOC+esDNA (81%), T4: FOC+esDNA (87%)). Bars represent means ± standard error (*n* = 3). Different lowercase letters denote significant differences among treatments (one-way ANOVA followed by Tukey’s HSD test, *p* < 0.05).

We quantified Foc abundance in banana corm and root tissues by qPCR to assess the disease control efficacy of each treatment. The treatments differed highly significantly in pathogen abundance: esDNA (87%) ≈ esDNA (81%) > esDNA (61%) > esDNA (30%) ≈ CK. Notably, esDNA derived from lower disease severity (30% and 61%) produced the strongest control, yielding Foc DNA levels in both corms and roots that fell into the lowest statistical subset, comparable to the healthy control (Figure 2A,B).

### 3.3. Differential Regulation of Osmotic Adjustment and Oxidative Stress by esDNA with Different Disease Indices

**Figure 3:**
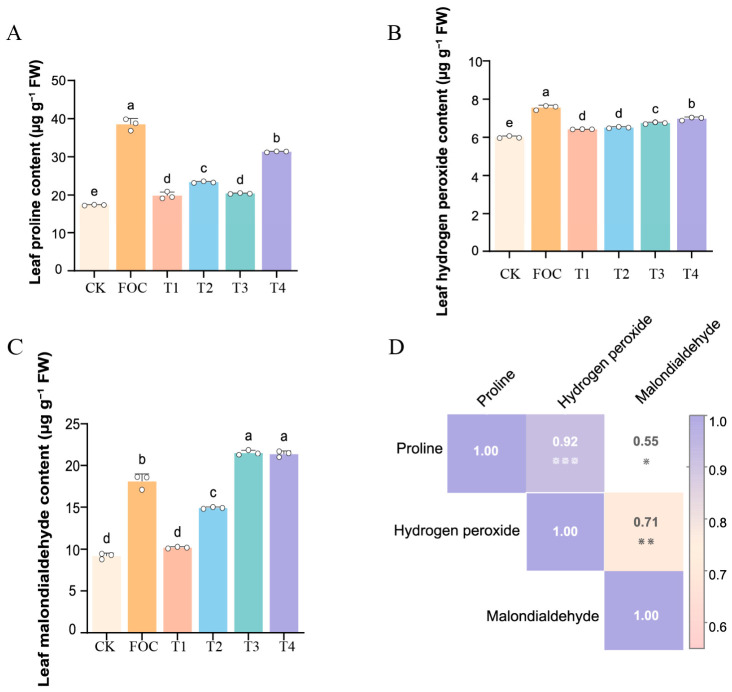
Regulatory effects of esDNA from leaves with different disease indices on osmotic adjustment and oxidative stress in banana leaves under Foc TR4 challenge. (**A**–**C**) Contents of proline (**A**), hydrogen peroxide (H_2_O_2_) (**B**), and malondialdehyde (MDA) (**C**) in banana leaves. Treatments: sterile water control (CK), Foc TR4 inoculation alone (Foc), and Foc TR4 inoculation combined with esDNA from leaves with disease indices of 30%, 61%, 81%, and 87% (T1: FOC+esDNA (30%), T2: FOC+esDNA (61%), T3: FOC+esDNA (81%). (**D**) Pearson correlation heatmap of proline, H_2_O_2_, and MDA. Values indicate correlation coefficients (r); asterisks represent significance levels (* *p* < 0.05, ** *p* < 0.01, *** *p* < 0.001). Data are means ± standard deviation (SD). Different lowercase letters indicate significant differences among treatments (Tukey’s HSD test, *p* < 0.05).

Proline content exhibited a clear gradient across treatments, with lower osmolyte accumulation in esDNA (30%) and esDNA (61%) groups (Figure 3A). Malondialdehyde (MDA) followed a similar pattern: no significant difference occurred between esDNA (30%) and CK, whereas MDA levels in esDNA (87%) and esDNA (81%) exceeded those in the Foc group, indicating the potential cytotoxicity of esDNA derived from highly diseased tissues (Figure 3B).

For H_2_O_2_ accumulation, esDNA (30%) and esDNA (61%) were significantly lower than in Foc and did not differ from CK, suggesting that esDNA from appropriately diseased sources maintains H_2_O_2_ at a signaling-favorable concentration and prevents oxidative damage from excessive ROS (Figure 3D).

Consistent with these physiological responses, Pearson correlation analysis revealed strong positive correlations between proline and H_2_O_2_ content (r = 0.92, *n* = 3, *p* < 0.001), and between MDA and H_2_O_2_ (r = 0.71, *n* = 3, *p* < 0.01), confirming coordinated induction of osmotic and oxidative stress responses under Foc challenge. Notably, proline and MDA were also significantly correlated (r = 0.55, *p* < 0.05), reinforcing that esDNA from highly diseased tissues exacerbated membrane lipid peroxidation and stress-related metabolite accumulation (Figure 3D).

### 3.4. esDNA Enhances Basal Resistance by Activating Phenylpropanoid Metabolism and Antioxidant Enzyme Systems

**Figure 4:**
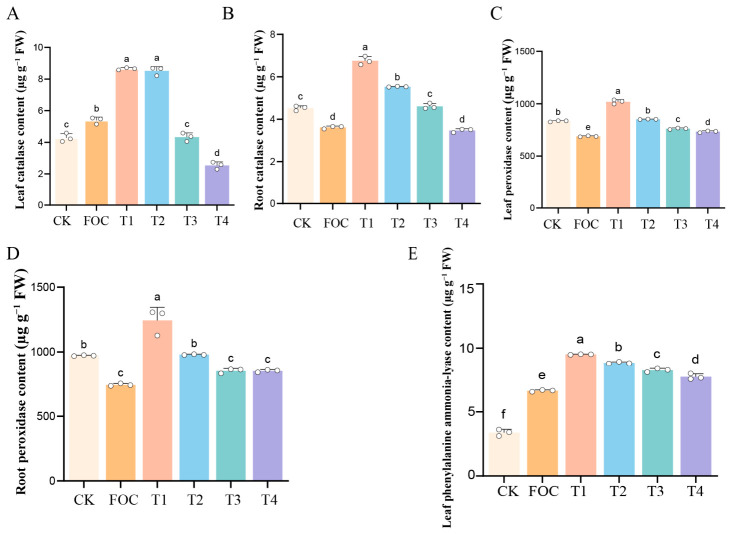
Modulation of antioxidant and defense-related enzyme activities in banana plants by esDNA derived from leaves with varying disease indices under Foc TR4 stress. (**A**–**D**) Activities of catalase (CAT) and peroxidase (POD) in leaf (**A**,**C**) and root (**B**,**D**) tissues. (**E**) Phenylalanine ammonia-lyase (PAL) activity in leaf tissues. Treatments: sterile water control (CK), Foc TR4 inoculation alone (Foc), and Foc TR4 inoculation combined with esDNA extracted from leaves with disease indices of 30%, 61%, 81%, or 87% (T1: FOC+esDNA (30%), T2: FOC+esDNA (61%), T3: FOC+esDNA (81%)). Data are means ± standard deviation (SD) (*n* = 3). Different lowercase letters indicate significant differences among treatments (Duncan’s multiple range test, *p* < 0.05).

PAL activity exhibited a clear induction gradient: Foc < esDNA (87%) ≈ esDNA (81%) < esDNA (61%) < esDNA (30%) ≈ CK. esDNA (61%) produced the strongest induction and further increased PAL activity compared with Foc, indicating that esDNA specifically and strongly activates the phenylpropanoid pathway, a core defense route (Figure 4E).

POD and CAT activities displayed organ specificity: both enzymes reached their highest activities in leaves and roots when treated with esDNA (30%) and esDNA (61%), whereas esDNA (87%) produced the lowest activities. Leaves were more responsive to treatments, indicating functional differentiation among organs in antioxidant defense. In summary, esDNA from mild and moderate disease sources enhances ROS-scavenging capacity, whereas esDNA from severely diseased tissues (87%) inhibits it (Figure 4A–D).

### 3.5. esDNA Modulates Pathogen-Induced Transcriptional Responses in Banana Leaves

**Figure 5:**
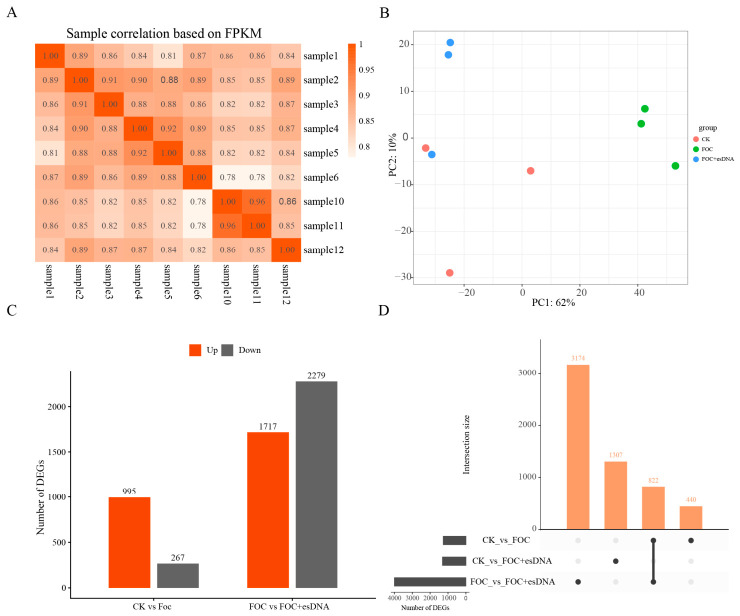
Transcriptomic profiling of banana leaves under different treatments. (**A**) Sample-to-sample correlation analysis among the three groups. (**B**) Principal component analysis (PCA) showing global transcriptional variation. (**C**) Numbers of differentially expressed genes (DEGs) identified in CK vs. FOC and FOC vs. FOC+esDNA comparisons. (**D**) UpSet plot illustrating the overlap of DEGs among CK vs. FOC, CK vs. FOC+esDNA, and FOC vs. FOC+esDNA comparisons.

Replicate samples were highly consistent, with correlation coefficients > 0.80, indicating reliable sequencing data suitable for downstream analyses (Figure 5A). Principal component analysis (PCA) showed clear separation among CK, FOC, and FOC+esDNA treatments; PC1 and PC2 accounted for 62% and 10% of the variance, respectively (Figure 5B), indicating substantial transcriptional differences among treatments. Differential expression analysis detected 1021 DEGs between CK and FOC and 3996 DEGs between FOC and FOC+esDNA (Figure 5C). Relative to CK, FOC induced 995 up-regulated genes, whereas 2279 genes were down-regulated in FOC+esDNA compared with FOC. These findings indicate that esDNA substantially modified pathogen-induced transcriptional responses. UpSet analysis showed that 822 DEGs were common to all three comparisons (CK vs. FOC, CK vs. FOC+esDNA, and FOC vs. FOC+esDNA) (Figure 5D), pointing to a shared transcriptional program responsive to both pathogen infection and esDNA treatment.

**Figure 6:**
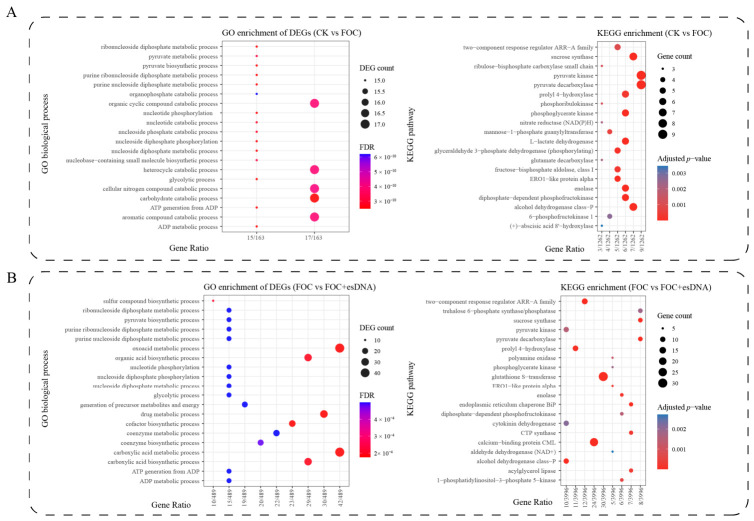
Functional enrichment analysis reveals biological processes and pathways affected by pathogen infection and esDNA treatment. (**A**) GO and KEGG enrichment analyses of DEGs between CK and FOC, showing biological processes and pathways associated with pathogen-induced responses. (**B**) GO and KEGG enrichment analyses of DEGs between FOC and FOC+esDNA, highlighting functional categories modulated by esDNA under infection conditions. Bubble size represents gene counts, and the color gradient corresponds to adjusted *p*-values (FDR).

GO biological process enrichment analysis revealed that genes responsive to pathogen infection were predominantly associated with primary metabolic and energy-related processes. These included the ribonucleoside diphosphate metabolic process, pyruvate metabolic process, pyruvate biosynthetic process, and various nucleotide metabolism-related pathways, such as the nucleoside diphosphate metabolic process and nucleotide phosphorylation. Additionally, several catabolic processes, including the organophosphate catabolic process and organic cyclic compound catabolic process, were significantly enriched. KEGG enrichment analysis further indicated that pathogen infection influenced pathways related to carbon metabolism, nitrogen assimilation, and signal transduction. Key enzymes such as pyruvate kinase, pyruvate decarboxylase, phosphoglycerate kinase, sucrose synthase, and nitrate reductase were identified within these pathways. Concurrently, regulatory components, including the two-component response regulator ARR-A family, were also enriched, suggesting the activation of hormone- and stress-related signaling pathways (Figure 6A). Collectively, these findings demonstrate that FOC infection induces extensive metabolic reprogramming and signaling activation in banana leaves.

Compared with pathogen treatment alone, esDNA application markedly altered metabolic and redox-related processes. GO enrichment indicated overrepresentation of pathways for sulfur compound biosynthesis, organic acid metabolism, nucleotide phosphorylation, and nucleoside diphosphate metabolism, reflecting a reconfiguration of cellular metabolic balance under esDNA. KEGG analysis showed enrichment of genes in carbohydrate metabolism, redox regulation, and stress signaling, including trehalose-6-phosphate synthase/phosphatase, sucrose synthase, polyamine oxidase, and glutathione S-transferase. Signaling-related genes, such as members of the two-component response regulator ARR-A family, also remained enriched, suggesting that esDNA modulates stress signaling rather than simply suppressing it (Figure 6B). Together, these results indicate that esDNA reshapes pathogen-induced transcriptional programs, with pronounced effects on metabolic homeostasis and oxidative stress regulation during infection.

**Figure 7:**
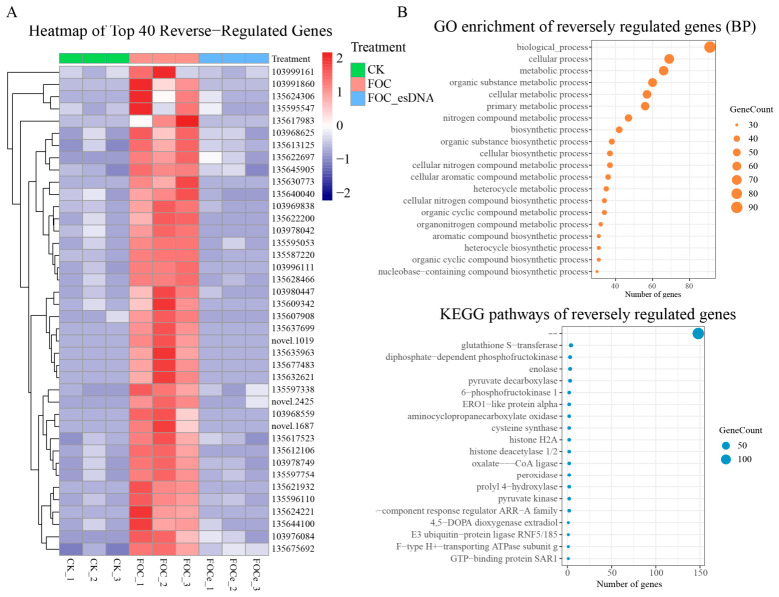
Reversely regulated genes under esDNA treatment counteract pathogen-induced transcriptional responses. (**A**) Heatmap showing expression patterns of reversely regulated genes (up-regulated in CK vs. FOC but down-regulated in FOC vs. FOC+esDNA). (**B**) GO and KEGG enrichment analyses of reversely regulated genes, revealing biological processes and pathways antagonistically modulated by esDNA during pathogen infection. Enrichment analysis was performed using the hypergeometric test with Benjamini–Hochberg multiple testing correction. Bubble size represents the number of genes enriched in each term, and the color gradient indicates the *p*-value. Only terms with *p* < 0.05 were considered statistically significant.

To further identify genes linked to esDNA’s regulatory effect during pathogen infection, we defined reverse-regulated genes as those significantly up-regulated in the CK vs. FOC comparison but down-regulated in the FOC vs. FOC+esDNA comparison. The heatmap revealed a coherent expression pattern across treatments (Figure 7A). These genes showed low expression in CK, were strongly induced by FOC infection, and were down-regulated after esDNA treatment. Clustering separated the three treatments, and biological replicates grouped together, indicating robust, reproducible transcriptional changes.

GO enrichment analysis showed that these genes were predominantly associated with core metabolic and biosynthetic processes, including cellular metabolic process, nitrogen compound metabolic process, organic cyclic compound metabolic process, and nucleobase-containing compound biosynthetic process (Figure 7B). These enriched terms reflect primary metabolism and anabolic activity.

KEGG enrichment analysis showed that reverse-regulated genes were concentrated in pathways of redox regulation and energy metabolism, including glutathione S-transferase, peroxidase, cysteine synthase, pyruvate kinase, pyruvate decarboxylase, and diphosphate-dependent phosphofructokinase. Several genes involved in transcriptional and post-translational control were also overrepresented, notably histone deacetylase, E3 ubiquitin-protein ligase, the GTP-binding protein SAR1, and members of the two-component response regulator ARR-A family.

Combined with the global enrichment patterns in Figure 7, these results indicate that esDNA treatment attenuates a subset of pathogen-induced transcriptional responses, particularly those linked to metabolic reprogramming and redox-related processes.

## 4. Discussion

By systematically comparing the regulatory effects of esDNA from banana tissues at different disease severities on the banana–Foc TR4 interaction, this study reveals a disease-stage-dependent activity of esDNA and provides a theoretical foundation for precise immune-regulation strategies based on DAMP signaling.

The present study shows that exogenously applied esDNA does not uniformly induce resistance. esDNA derived from mildly diseased tissues produced the strongest protective effects across multiple key indices. It most effectively suppressed colonization and proliferation of Foc TR4 in banana root and corm tissues (Figure 2) and best preserved plant growth under pathogenic stress, particularly corm biomass accumulation (Figure 1). At the physiological level, esDNA from mildly diseased tissues alleviated osmotic stress by reducing excessive proline accumulation (Figure 3A) and limited oxidative damage by markedly lowering MDA and H_2_O_2_ contents (Figure 3B,C) induced by pathogen infection. It also specifically and strongly activated the phenylpropanoid pathway enzyme PAL and the antioxidant enzymes POD and CAT (Figure 4). These results indicate that esDNA from mildly diseased tissues may act as an alert signal, potentially initiating a moderate and coordinated defense response. Further studies are needed to confirm the specific signaling pathways involved. This process controls disease development while avoiding resource depletion and self-inflicted damage caused by excessive immune responses. The finding supports the defense trade-off theory and suggests that appropriate DAMP signals enable an optimized balance between defense activation and growth maintenance in plants [8,10].

In contrast to esDNA derived from mildly diseased tissues, esDNA from severely diseased tissues nearly lost its protective efficacy. It not only failed to limit pathogen proliferation but also aggravated cellular oxidative damage and membrane lipid peroxidation for certain indices (Figure 3). This shift from protective to ineffective or even harmful activity directly demonstrates that esDNA immune function depends on the pathological state of the source tissue. We propose that as tissue necrosis advances, the extracellular DNA pool undergoes marked changes in fragmentation, oxidative modification (for example, 8-OHdG), and the composition of co-released degradation products such as ATP, glutamate, and other DAMPs. Highly fragmented or modified DNA may engage different pattern recognition receptors (PRRs), be excessively consumed or rendered inert as part of the canonical “self” signal, and thus provoke aberrant or nonfunctional immune signaling. At high concentrations, oxidative byproducts may also produce direct cytotoxic effects [20,21,24]. These findings address the limitations of prior work that mainly used homogeneous DNA from healthy tissues, emphasize the need to evaluate DAMP function dynamically during natural disease progression, and expand the damaged-self recognition framework to encompass a continuous and more complex pathological spectrum [14].

Transcriptomic analysis offers mechanistic insight at the molecular level to account for the observed phenotypic and physiological changes. Compared with pathogen stress alone, esDNA from mildly diseased tissues elicited extensive transcriptional reprogramming and specifically reversed the expression of a subset of pathogen-induced genes (Figure 5). Functional enrichment analysis showed that these esDNA-reversely regulated genes, and the pathways globally modulated by esDNA treatment, were significantly enriched for carbohydrate metabolism, nucleotide metabolism, glutathione metabolism, and peroxidase activity (Figure 6). These findings indicate that Foc TR4 infection pushes host cells into a hypermetabolic, high-stress state. Rather than broadly amplifying immune responses, protective esDNA reprograms this pathological metabolic state and redirects resources from excessive, potentially disordered stress metabolism toward core pathways that sustain redox homeostasis and basal resistance. This transcriptional mechanism of homeostasis remodeling aligns with the physiological phenotypes observed (reduced oxidative damage, preserved growth) and explains how esDNA establishes an economical, long-lasting state of systemic acquired resistance (SAR) or immune priming [17,18,19].

All treatments markedly inhibited the architectural development of banana root systems (Figure 1). This inhibition is unlikely to result from simple resource trade-offs, because esDNA from mildly diseased tissues suppressed root growth while strongly promoting aboveground growth and disease control. The response may therefore represent an adaptive strategy: upon perceiving pathogen- or DAMP-derived signals in roots, the plant actively reduces exploratory root growth in pathogen-enriched soil and reallocates resources to aboveground preservation, storage organ development, and localized chemical defenses in roots, for example by enhancing antioxidant enzyme activity. Such a contraction-defense strategy could confer ecological advantages by limiting pathogen contact and reducing the number of potential infection sites [4,17,30].

Collectively, this study opens a promising avenue for ecologically sound disease management using endogenous plant immune signals. Future research should isolate and identify the key active components within esDNA mixtures at different disease stages; identify putative receptors and dissect downstream signaling pathways in banana [19]; and evaluate the stability, persistence, and interactions of active esDNA with soil microbiomes in soil microecosystems.

Although our results provide new insights into the function of esDNA in plant defense, several limitations of this study should be acknowledged. First, our experiment lacked an esDNA-only treatment, which hinders the separation of direct esDNA effects from esDNA–Foc interactive responses. Based on comparisons between the Foc and Foc+esDNA groups, together with phenotypic and physiological evidence, we infer that the observed physiological changes were primarily derived from esDNA regulation. Nevertheless, this limitation is acknowledged, and future studies will include an esDNA-only control to clarify its direct functions. Second, while our physiological and transcriptomic data suggest that esDNA from mildly diseased tissues may enhance resistance by modulating metabolic and redox homeostasis, direct causal evidence linking specific transcriptional changes to the protective phenotype is not provided. Third, the proposed role of esDNA as a DAMP signal is inferred from phenotypic and transcriptional responses; receptor identification and downstream signaling validation are required in future studies. Fourth, all experiments were conducted under controlled hydroponic conditions; therefore, validation under field conditions with natural soil and microbiota will be necessary to assess the practical applicability of esDNA-based immune priming.

## 5. Conclusions

This study elucidates the disease-stage-dependent immune regulatory role of esDNA in banana–Foc TR4 interactions. We demonstrated that esDNA derived from mildly diseased tissues serves as a potent immune elicitor, effectively limiting *Fusarium* wilt without incurring significant growth penalties. In contrast, esDNA from severely diseased tissues exhibits diminished protective activity due to altered signaling properties. These findings enhance the understanding of plant DAMP signaling within dynamic pathological contexts and underscore the potential of functional esDNA as a foundation for the development of novel plant-derived green immune inducers.

## Data Availability

The raw RNA-sequencing (RNA-seq) data generated in this study have been deposited in the Genome Sequence Archive (GSA) at the Beijing Institute of Genomics (BIG) Data Center, Chinese Academy of Sciences, and are publicly accessible under BioProject accession number CRA063172 at https://ngdc.cncb.ac.cn/gsa/browse/CRA042140 accessed on 23 April 2025.

## References

[1] Ploetz R.C. (2015). *Fusarium* Wilt of Banana. Phytopathology.

[2] Dita M., Barquero M., Heck D., Mizubuti E.S.G., Staver C.P. (2018). *Fusarium* Wilt of Banana: Current Knowledge on Epidemiology and Research Needs Toward Sustainable Disease Management. Front. Plant Sci..

[3] Dong X., Xiong Y., Ling N., Shen Q., Guo S. (2014). Fusaric acid accelerates the senescence of leaf in banana when infected by *Fusarium*. World J. Microbiol. Biotechnol..

[4] Li C., Yang J., Li W., Sun J., Peng M. (2017). Direct Root Penetration and Rhizome Vascular Colonization by *Fusarium oxysporum* f. sp. *cubense* are the Key Steps in the Successful Infection of Brazil Cavendish. Plant Dis..

[5] Liu X., Arshad R., Wang X., Li W.-M., Zhou Y., Ge X.-J., Huang H.-R. (2023). The phased telomere-to-telomere reference genome of *Musa acuminata*, a main contributor to banana cultivars. Sci. Data.

[6] Bubici G., Kaushal M., Prigigallo M.I., Gómez-Lama Cabanás C., Mercado-Blanco J. (2019). Biological Control Agents Against *Fusarium* Wilt of Banana. Front. Microbiol..

[7] Ruiz C., Nadal A., Montesinos E., Pla M. (2018). Novel Rosaceae plant elicitor peptides as sustainable tools to control *Xanthomonas arboricola* pv. *pruni* in *Prunus* spp.. Mol. Plant Pathol..

[8] Fernández-Calvo P., López G., Martín-Dacal M., Aitouguinane M., Carrasco-López C., González-Bodí S., Bacete L., Mélida H., Sánchez-Vallet A., Molina A. (2024). Leucine rich repeat-malectin receptor kinases IGP1/CORK1, IGP3 and IGP4 are required for *Arabidopsis* immune responses triggered by β-1,4-D-Xylo-oligosaccharides from plant cell walls. Cell Surf..

[9] Jing Y., Zhao F., Lai K., Sun F., Sun C., Zou X., Xu M., Fu A., Sharifi R., Chen J. (2024). Plant elicitor Peptides regulate root hair development in *Arabidopsis*. Front. Plant Sci..

[10] Matzinger P. (2002). The Danger Model: A Renewed Sense of Self. Science.

[11] Chen G.Y., Nuñez G. (2010). Sterile inflammation: Sensing and reacting to damage. Nat. Rev. Immunol..

[12] Huang M.L., Zhang Y., Wang Y., Xie J.T., Cheng J.S., Fu Y.P., Jiang D.H., Yu X., Li B. (2022). Active DNA demethylation regulates MAMP-triggered immune priming in Arabidopsis. J. Genet. Genom..

[13] Barbero F., Guglielmotto M., Capuzzo A., Maffei M.E. (2016). Extracellular Self-DNA (esDNA), but Not Heterologous Plant or Insect DNA (etDNA), Induces Plasma Membrane Depolarization and Calcium Signaling in Lima Bean (*Phaseolus lunatus*) and Maize (*Zea mays*). Int. J. Mol. Sci..

[14] Duran-Flores D., Heil M. (2018). Extracellular self-DNA as a damage-associated molecular pattern (DAMP) that triggers self-specific immunity induction in plants. Brain Behav. Immun..

[15] Duran-Flores D., Heil M. (2016). Sources of specificity in plant damaged-self recognition. Curr. Opin. Plant Biol..

[16] Mazzoleni S., Cartenì F., Bonanomi G., Senatore M., Termolino P., Giannino F., Incerti G., Rietkerk M., Lanzotti V., Chiusano M.L. (2015). Inhibitory effects of extracellular self-DNA: A general biological process?. New Phytol..

[17] Li C.H., Wang K.T., Zou Y.Y., Lei C.Y., Chen Z.X., Zheng Y.H. (2023). Extracellular self-DNA induced a PTI-related local defence against *Rhizopus* rot in postharvest peach fruit. Postharvest Biol. Technol..

[18] Zhou X., Gao H., Zhang X., Khashi u Rahman M., Mazzoleni S., Du M., Wu F. (2023). Plant extracellular self-DNA inhibits growth and induces immunity via the jasmonate signaling pathway. Plant Physiol..

[19] Gamir J., Vega-Muñoz I., Rassizadeh L., Heil M. (2025). On the quest for undiscovered plant DNA receptors. Trends Plant Sci..

[20] Vega-Muñoz I., Feregrino-Pérez A.A., Torres-Pacheco I., Guevara-González R.G. (2018). Exogenous fragmented DNA acts as a damage-associated molecular pattern (DAMP) inducing changes in CpG DNA methylation and defence-related responses in *Lactuca sativa*. Funct. Plant Biol..

[21] Tjia T.O.S., Meitha K., Septiani P., Awaludin R., Sumardi D. (2023). Extracellular self-DNA induces local inhibition of growth, regulates production of reactive oxygen species, and gene expression in rice roots. Biol. Plant..

[22] Kechin A., Boldyreva D., Borobova V., Boyarskikh U., Scherbak S., Apalko S., Makarova M., Mosyakin N., Kaftyreva L., Filipenko M. (2021). An inexpensive, simple and effective method of genome DNA fragmentation for NGS libraries. J. Biochem..

[23] Ferrusquía-Jiménez N.I., Chandrakasan G., Torres-Pacheco I., Rico-Garcia E., Feregrino-Perez A.A., Guevara-González R.G. (2021). Extracellular DNA: A Relevant Plant Damage-Associated Molecular Pattern (DAMP) for Crop Protection Against Pests—A Review. J. Plant Growth Regul..

[24] Carbajal-Valenzuela I.A., Guzmán-Cruz R., González-Chavira M.M., Medina-Ramos G., Serrano-Jamaica L.M., Torres-Pacheco I., Vázquez L., Feregrino-Pérez A.A., Rico-García E., Guevara-González R.G. (2022). Response of Plant Immunity Markers to Early and Late Application of Extracellular DNA from Different Sources in Tomato (*Solanum lycopersicum*). Agriculture.

[25] Ullah S., Mostert D., Serfontein K., Viljoen A. (2021). The Survival and Treatment of *Fusarium oxysporum* f. sp. *cubense* in Water. J. Fungi.

[26] Paramalingam P., Baharum N.A., Abdullah J.O., Hong J.K., Saidi N.B. (2023). Antifungal Potential of *Melaleuca alternifolia* against Fungal Pathogen *Fusarium oxysporum* f. sp. *cubense* Tropical Race 4. Molecules.

[27] Bragard C., Baptista P., Chatzivassiliou E., Di Serio F., Gonthier P., Jaques Miret J.A., Justesen A.F., MacLeod A., Magnusson C.S., EFSA Panel on Plant Health (PLH) (2022). Pest categorisation of *Fusarium oxysporum* f. sp. *cubense* Tropical Race 4. EFSA J..

[28] Liñares-Blanco J., Fernandez-Lozano C., Seoane J.A., López-Campos G. (2022). Machine Learning Based Microbiome Signature to Predict Inflammatory Bowel Disease Subtypes. Front. Microbiol..

[29] Fang J., Ma J., Wen T., Niu G., Wei S., Su S., Yi L., Cheng Y., Yuan J., Zhao X. (2025). Cry for help from rhizosphere microbiomes and self-rescue strategies cooperatively alleviate drought stress in spring wheat. Soil Biol. Biochem..

[30] Wu Y., Huang B., Peng X., Zhang J. (2021). Development of an in vitro hydroponic system for studying the interaction between banana plantlet and *Fusarium oxysporum* f. sp. *cubense*. Plant Cell Tissue Organ Cult..

[31] Ciccoritti R., Manganiello R., Antonucci F., Ceccarelli D. (2023). Interactive Effect of Cultivars, Crop Years and Rootstocks on the Biochemical Traits of *Prunus persica* (L.) Batsch Fruits. Plants.

[32] Miler N., Tymoszuk A., Woźny A., Michalik T., Wiśniewska J., Kulus D. (2024). Microbiological Biostimulants in the Improvement of Extended Storage Quality of In Vitro-Derived Plants of Popular Ornamental Perennials. Agronomy.

[33] Jiménez-Fernández D., Montes-Borrego M., Navas-Cortés J.A., Jiménez-Díaz R.M., Landa B.B. (2010). Identification and quantification of *Fusarium oxysporum* in planta and soil by means of an improved specific and quantitative PCR assay. Appl. Soil Ecol..

[34] Anuradha C., Chandrasekar A., Backiyarani S., Thangavelu R., Uma S., Selvarajan R. (2024). Dataset from transcriptome profiling of *Musa* resistant and susceptible cultivars in response to *Fusarium oxysporum* f. sp. *cubense* race1 and TR4 challenges using Illumina NovaSeq. Data Brief.

[35] Love M.I., Huber W., Anders S. (2014). Moderated estimation of fold change and dispersion for RNA-seq data with DESeq2. Genome Biol..

[36] Wu T., Hu E., Xu S., Chen M., Guo P., Dai Z., Feng T., Zhou L., Tang W., Zhan L. (2021). clusterProfiler 4.0: A universal enrichment tool for interpreting omics data. Innovation.

[37] Marini F., Binder H. (2019). pcaExplorer: An R/Bioconductor package for interacting with RNA-seq principal components. BMC Bioinform..

